# Long Non-Coding RNA and Acute Leukemia

**DOI:** 10.3390/ijms20030735

**Published:** 2019-02-09

**Authors:** Gabriela Marisol Cruz-Miranda, Alfredo Hidalgo-Miranda, Diego Alberto Bárcenas-López, Juan Carlos Núñez-Enríquez, Julian Ramírez-Bello, Juan Manuel Mejía-Aranguré, Silvia Jiménez-Morales

**Affiliations:** 1 Programa de Doctorado, Posgrado en Ciencias Biológicas, Universidad Nacional Autónoma de México, Mexico City 04510, Mexico; gmcm611@hotmail.com (G.M.C.-M.); d.a.barcenas@outlook.com (D.A.B.-L.); 2 Laboratorio de Genómica del Cáncer, Instituto Nacional de Medicina Genómica, Mexico City 14610, Mexico; ahidalgo@inmegen.gob.mx; 3 Unidad de Investigación Médica en Epidemiología Clínica, UMAE Hospital de Pediatría “Dr. Silvestre Frenk Freund”, Centro Médico Nacional Siglo XXI, Instituto Mexicano del Seguro Social, Mexico City 06720, Mexico; jcarlos_nu@hotmail.com; 4 Unidad de Investigación en Enfermedades Metabólicas y Endócrinas, Hospital Juárez de México, Mexico City 07760, Mexico; dr.julian.ramirez.hjm@gmail.com; 5 Coordinación de Investigación en Salud, Instituto Mexicano del Seguro Social, Mexico City 06720, Mexico

**Keywords:** long non-coding RNAs, cancer, acute leukemia, therapeutic targets

## Abstract

Acute leukemia (AL) is the main type of cancer in children worldwide. Mortality by this disease is high in developing countries and its etiology remains unanswered. Evidences showing the role of the long non-coding RNAs (lncRNAs) in the pathophysiology of hematological malignancies have increased drastically in the last decade. In addition to the contribution of these lncRNAs in leukemogenesis, recent studies have suggested that lncRNAs could be used as biomarkers in the diagnosis, prognosis, and therapeutic response in leukemia patients. The focus of this review is to describe the functional classification, biogenesis, and the role of lncRNAs in leukemogenesis, to summarize the evidence about the lncRNAs which are playing a role in AL, and how these genes could be useful as potential therapeutic targets.

## 1. Introduction

Leukemia is a group of hematological malignancies characterized by an oligoclonal expansion of abnormally differentiated, and sometimes poorly differentiated hematopoietic cells which infiltrate the bone marrow, and could also invade the blood and other extramedullary tissues. In general, AL can be divided into acute or chronic, and lymphoid or myeloid, according to their progression and affected lineage, respectively. Thus, we can identify the following subtypes: acute lymphoblastic leukemia (ALL), chronic lymphoblastic leukemia (CLL), acute myeloid leukemia (AML), and chronic myeloid leukemia (CML). AL is the main type of cancer in children worldwide [1,2]. In recent years, it has reported a trend of increase in the incidence AL; notwithstanding, the causes are still unclear. Studies conducted to identify the etiology of this disease have reported that a genetic background interacting with environmental factors (i.e., high doses of ionizing radiation, infections, parental occupational exposures, etc.) could explain this phenomenon [3]; however, the molecular mechanisms involved are not fully understood. To date, growing data have shown that different non-coding RNAs (ncRNAs) might be the link between the genome and the environment because they are closely related to normal physiological and pathological processes [4,5]. ncRNAs, also known as non-protein-coding RNAs (npcRNAs), non-messenger RNAs (nmRNAs) or functional RNAs (fRNAs), are functional RNA molecules which are not translated into proteins [6]. These RNAs consist of several distinct families which include microRNAs (miRNAs), small nuclear RNAs (snRNAs), PIWI-interacting RNAs (piRNAs), and long non-coding RNAs (lncRNAs), among others. LncRNAs are one of the most studied ncRNA types, and play an important role as gene expression modulators at the epigenetic, transcriptional, and post-transcriptional level. In fact, it has been suggested that various miRNAs and lncRNAs could act as tumor suppressors genes or oncogenes, because they regulate directly or indirectly the expression of genes involved in molecular mechanisms as cell proliferation/differentiation, apoptosis, and metastasis [4,5]. In comparison with miRNAs, the lncRNAs are more numerous and represents the 41% of the overall ncRNAs. Over the last years, massive technological tools have been useful to increase the knowledge about lncRNAs that are abnormally expressed or mutated in AL and the list of relevant lncRNAs in leukemogenesis is growing rapidly. Moreover, it has reported a distinctive lncRNAs expression signature associated with AL prognosis, suggesting the potential application of these genes to make treatment decisions. Here, we review the most recent findings about lncRNAs in AL pathogenesis and their role as potential biomarkers. We also are pointing out the lncRNAs as promising druggable molecules in the development of new treatments for leukemia [7]. An electronic search strategy using the biomedical database of the National Center for Biotechnology Information (NCBI) was conducted. Studies that combined the keywords lncRNAs with acute leukemia, or acute lymphoblastic leukemia, or acute myeloid leukemia or hematopoiesis were enclosed.

## 2. Genetic Features of Acute Leukemia

AL has been recognized as a highly genetically heterogeneous disease, where chromosomal abnormalities, either numerical (hyperdiploidy and hypodiploidy) or structural alterations (translocations, amplifications, DNA copy number alterations, insertions/deletions, and punctual mutations) are usually observed; thus, these alterations are the hallmarks of the leukemic cells and represent the major class of oncogenic drivers to the disease. Indeed, due to the fact many childhood ALL cases carry specific fusion genes (*MLL* gene fusions, *ETV6*/*RUNX1*, *E2A*/*PBX1*, etc.) and *AML* (*AML1*/*ETO*, *PML*/*RARα*, *CBFβ*/*MYH11*, etc.), this gives more evidence that childhood AL is initiated in utero during fetal hematopoiesis [8]. In addition to the numerical alterations and common targets of translocations in ALL, this disease is characterized by mutations in transcriptional factors (*AML1*, *ETS*, *PAX5*, *IKZF1*, *EBF1*, *ETV6*, and *STAT*), suppressor genes (*TP53*, *RB1*, *CDKN2A/CDKN2B*, etc.), oncogenes (*ABL1*, *ABL2*, *CSF1R*, *JAK2*, *PDGFRB*, and *CRLF2*), B lymphoid cell differentiators (*IKZF1*, *TCF3*, *EBF1*, *PAX5*, and *VPREB1)*, chromatin remodelers, or epigenetic modifiers (*DNMT3A*, *CREBBP*, *MLL2*, *NSD2*, *EP300*, *ARID1A*, *TET2*, and *CHD6*) [9,10,11,12]. Data from the St. Jude/Washington Pediatric Cancer Genome Project (PCGP), that has characterized pediatric cancer genomes by whole-genome or whole-exome sequencing, revealed that the somatic mutation rate in childhood ALL ranges from 7.30 × 10^−8^ per base [13]. In spite of the fact that chromosomal changes detectable by cytogenetic techniques are present in nearly 75% of the precursor B (pre-B) cell ALL cases, the gene expression profiling and genome-wide sequencing analyses have showed that B cell leukemogenesis is more complex [14]. Meanwhile, mutations in *nRAS*, *RUNX1*, *FLT3*, *KIT*, etc., abnormalities of DNA methylation, biogenesis of ribosomes, activated signaling pathways, myeloid transcription factors, chromatin remodeling, and cohesion complex processes are very common in AML [15].

The discovery of frequent mutations in epigenetic modifiers genes in AL show that epigenetic alterations also play a critical role in leukemogenesis. In this regard, it is known that most of the genes involved in epigenetic process do not code for proteins, and many of them are classified as lncRNAs, which regulate gene expression through different mechanisms.

## 3. LncRNAs Characteristics

lncRNAs comprise a highly functionally heterogeneous group of RNA molecules with sizes are greater than 200 nucleotides, and, as all the mRNAs usually have more than one exon, most of them are transcribed by RNA polymerase II (RNA pol II), are capped, may be polyadenylate, and can be located within the nucleus or cytoplasm. LncRNAs genes differ from mRNAs because lncRNAs lack protein-coding potential, are mostly expressed in low levels, and show poor species conservation compared to protein-coding genes (mRNAs). Additionally, lncRNAs display tissue-specific and development stage-specific expression showing their important role in cell differentiation mechanisms [16].

The number of lncRNAs is larger than the number of protein-coding RNAs. To date, the GENCODE project lncRNAs catalog consists of 15,779 transcripts (there are potentially more than 28,000 distinct transcripts) in the human genome (https://www.gencodegenes.org); nevertheless, this number could increase, since many primary long non-coding transcripts are often processed into smaller ncRNAs [17]. ncRNA detection led to a solution for the G-value paradox that states that there is no correlation between the amount of coding genes and the complexity of the organism, while we observe a correlation between the complexity of the organism and the ratio of the number of non-coding genes to total genomic DNA. Nowadays, cumulative evidence exhibits that lncRNAs are relevant players in many cellular processes either in physiological as well as pathological conditions. In cancer, the lncRNAs could have oncogenic function and tumor suppressive function since they have been found as upregulated or downregulated in several types of tumors in comparison to healthy tissues [18].

## 4. Biogenesis and Classification

It has hypothesized that most of lncRNAs are originated from (1) the incorporation of the fragments of original protein-coding genes; (2) juxtaposition of two transcribed and previously well-separated sequence regions of chromosomes giving rise a multi-exon ncRNA; (3) duplication of non-coding genes through retrotransposition; (4) tandem duplication events of neighboring repeats within a ncRNA; and (5) insertion of transcription factor, which is inserted into a sequence.

LncRNAs are transcribed and processed by the RNA pol II transcriptional machinery, thus many of them undergo post-transcriptional modifications such as 5′ capping, splicing, and polyadenylation. Nevertheless, there are also nonpolyadenylated lncRNAs that derive from RNA pol III promoters and snoRNA-related lncRNAs (sno-lncRNAs) expressed from introns via the snoRNP machinery (with the supplementary production of two snoRNAs). LncRNAs have been mapped into a wide range of regions, including coding and non-coding regions (intergenic regions, promoters, enhancers, and introns) [19,20,21,22,23,24,25,26,27].

**Figure 1:**
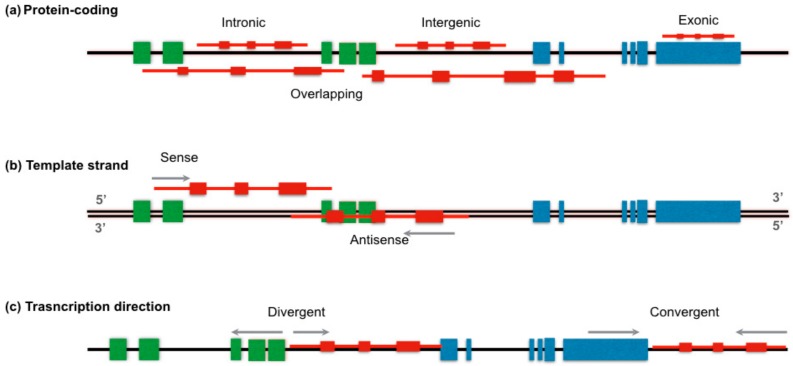
Positional classification of the long non-coding RNAs (lncRNA). Carton displays the LncRNA (red) classification base on (**a**) the location between two coding genes (intronic, exonic, intergenic, or overlapping), (**b**) the template strand (sense, antisense), and (**c**) transcription direction when coding genes and lncRNA are transcribed in the same strand (divergent, convergent). Gray arrow indicates in which direction transcription is proceed. Green and blue boxes represent exons of two different genes.

To date, there is not a unique system to classify lncRNAs; however, different classifications have been proposed based on their size, genome localization, RNA mechanism of action, and function [28]. According to their location (Figure 1a), orientation (Figure 1b), and transcription direction (Figure 1c) relative to protein-coding genes, an lncRNA can be placed into one or more broad categories. Thus, lncRNAs can be intronic, when they lie into a intron of a second transcript (*COLDAIR*, located in the first intron of the flowering repressor locus C or *FLC*), intergenic (lincRNA) if it is located between two genes without any overlap at least 5 kb from both sides (exemplified by *H19*, *XIST*, and *lincRNA-p21*), exonic if lncRNA is encoded within a exon, or overlapping, which includes those lncRNA located within one or two genes [4,13,29,30]. Based on the orientation, lncRNAs can be transcribed from either the same strand or antisense in a divergent or convergent manner. LncRNAs can be also classified as enhancer-associated RNAs (eRNAs) and promoter-associated long RNAs (or PROMPTs) if they are produced from enhancer or promoter regions, respectively [31].

Although lncRNAs show a spatiotemporal expression pattern during proliferation, differentiation, and cell death; these genes are classified based on their function as guide, decoy, signaling, scaffold, or enhancer lncRNAs [32]. Guide lncRNAs interact with transcription factors or proteins and recruit them to their gene target or their genomic loci regulating downstream signaling events and gene expression. Decoy lncRNAs mimic and compete with their consensus DNA-binding motifs for binding nuclear receptors or transcriptional factors in the nucleus, facilitating gene activation or silencing. These genes can also “sponge” proteins such as chromatin modifiers, adding an extra level transcriptome regulation. Signaling lncRNAs are associated with signaling pathways to regulate transcription in response to various stimuli. Scaffold lncRNAs act as a central platform where many protein complexes tying and get directed to specific genomic loci or target gene promoter [17]. Enhancer lnRNAs are *cis*-encoded DNA elements that bind with mediator complex to regulate transcription genes located within their own chromosome (Table 1) [33]. However, this classification is too simple to cover the whole lncRNAome, cases such as pseudogenes and telomerase RNA (*TERC*) still lie outside the list [20,32].

In terms of size, lincRNAs often range from hundreds of nucleotides to several kilobases [20]. Nevertheless, there are exceptionally long lncRNAs (macroRNAs) and very long intergenic non-coding RNAs (vlincRNAs), stretching 10 kb and 1 Mb, respectively [30].

In addition, lncRNAs have regulatory roles in gene expression at both, the transcriptional, and post-transcriptional levels in mostly biological mechanisms and pathophysiological processes. These molecules can regulate the expression of neighboring genes (*cis*) or affect genes located at different chromosomes (*trans*) [38]. In this way, lncRNAs can regulate gene expression via transcription factor and chromatin-modifiers complex recruitment to their DNA targets, acting as enhancers to activate genes, as part of the heterogeneous nuclear ribonucleoprotein (hnRNP) complex, interacting with RNA and DNA by base paring, etc. [38].

## 5. LncRNAs in Normal Hematopoiesis

Hematopoietic cell lineage differentiation involves the regulation of gene expression at different levels that can occur to activate lineage specific genes and repress those genes that are not specific to that lineage. This activation/suppression is mediated by transcription factors and chromatin remodeling that act as determinants of the intrinsic cell lineage. However, these factors are reactivated in different lines and stages of differentiation, so that the choice of the final lineage reflects the particular combination of elements interacting in a certain stage of cell differentiation [39]. LncRNAs are involved in regulating different steps in hematopoiesis, immune system development, and activation. In fact, several lncRNAs have been identified in the blood cells either in animal models or human samples. For example, over 1109 poliA+ lncRNAs were detected in murine megakaryocytes, erythroblast, and megakaryocyte-erythroid precursors, of which 15% are expressed in humans [40]. The Eosinophil Granule Ontogeny (*EGO*) was one of the first lncRNAs related with the human normal hematopoiesis process. *EGO* is nested within an intron of inositol triphosphate receptor type 1 (*ITPR1*) and was found to be highly expressed in human bone marrow and in mature eosinophils. Despite that the molecular mechanism of their actions is not well known, experimental evidences show that *EGO* is involved in the eosinophil differentiation of CD34+ hematopoietic progenitor cells by regulating eosinophil granule protein expression at the transcription level [41]. *PU.1-As*, which is antisense to the master hematopoietic transcriptional factor *PU.1*, negatively regulates the expression of *PU.1*, repressing myeloid cells and B cells differentiation [42]. Other examples include dendritic cell-specific lncRNA (*lnc*-*DC*), non-coding RNA repressor of *NFAT* (*NRON*), and *lincRNA-Cox2*. *lnc*-*DC* was identified from extensive profiling of lncRNAs expression during differentiation of monocytes into dendritic cells (DCs). Mechanistic studies suggest that *lnc*-*DC* contributes to prevent *STAT3* (signal transducer and activator of transcription 3) dephosphorylation by Src homology region 2 domain-containing phosphatase-1 (*SHP1*) by directly binding to *STAT* in the cytoplasm [43]. *NRON* plays a relevant role in the adaptive immune response through sequestering transcription factors in the cytoplasm, such as the nuclear factor of activated T cells (NFAT). *LincRNA-Cox2* contributes with the regulation of the innate immune response by repressing the expression of critical immune-response regulators and by the coordinating the assembly, location and orientation of the complexes that specify the cellular fate [39].

Studying twelve distinct blood cell population purified by multicolor flow cytometry, Schwarzer et al. [44] established a human ncRNA hematopoietic expression atlas per blood cell population, finding *LINC00173*, *LINC000524*, *RP11-1029J19*, and *HOTAIRM1* among the lncRNAs that characterize cells of the different human blood lineages. *LINC00173* exhibited the most specific expression, with critical regulatory circuits involved in blood homeostasis and myeloid differentiation. In vitro models showed that suppression of *LINC00173* in human CD34+ hematopoietic stem and progenitor cells (HSPCs) specifically affects granulocyte differentiation and decreases its phagocytic capacity (which is associated with perturbed maturation). Additional studies reported that *LINC00173* is highly expressed in granulocytes [45]. *H19*, *XIST*, *lncHSC-1*, and *lncHSC-2*, which maintain long-term hematopoietic stem cell (HSC) quiescence and self-renewal, have also been involved in normal hematopoiesis [46].

## 6. LncRNAs in Acute Leukemia

Although many studies have implicated lncRNAs in many cancer types, little is known about the functional impact of lncRNAs in AL etiology, progression, and treatment response [44]. Several lncRNAs have been reported to be exclusively involved in specific ALL lineages but few of these are abnormally expressed in ALL and AML [47,48]. For instance, *CASC15*, involved in cellular survival proliferation and the expression of *SOX4* (*cis* regulation), was detected to be upregulated in t(12;21) (p13;q22) (*ETV6/RUNX1*) B cell ALL and in AML patients with the (8;21) translocation. In both cases, upregulation of *CASC15* was associated with a good prognosis [48]. To date, a large number of lncRNAs have been identified in AL; however, their molecular mechanisms remains elusive. Table 2 includes some examples of lncRNAs which have been reported as implicated in acute leukemia in children [49,50,51,52,53,54,55,56,57,58,59,60,61,62,63,64,65,66,67,68,69,70,71,72,73,74,75,76,77].

## 7. LncRNAs in Acute Myeloid Leukemia

Regarding the association between lncRNA and hematopoietic cancer, AML has been the most investigated, and has been reported to be an important lncRNA in the biological and pathological processes of the disease. For example, insulin-like growth factor type I receptor antisense imprinted non-protein RNA (*IRAIN*), which is transcribed antisense to insulin-like growth factor type I receptor (*IGF1R*) gene, is downregulated in leukemia cell lines and in patients with high-risk AML. *IRAIN* is involved in the formation of a long-range intrachromosomal interaction between the *IGF1R* promoter and a distant intragenic enhancer [49]. *ZNF571-AS1* is another lncRNA that has been suggested as a relevant player in AML. Based on co-expression correlation analysis across all AML samples with *lncRNA–lncRNA* pairs, this lncRNA was identified as potential regulator of the Janus Kinase (JAK)/signal transducer and activator of transcription (STAT) 5A and tyrosine-protein kinase Kit (KIT) expression. Thus their participation in AML was suggested via the JAK/STAT signaling pathway [69]. As well, Urothelial carcinoma-associated 1 (*UCA1*), an oncofetal gene that has been involved in embryonic development and carcinogenesis, was found to be upregulated in myeloid cell lines promoting cell viability, migration, invasion, and apoptosis processes [78,79,80]. A significant upregulation of *UCA1* expression in AML with *CEBPA* (a crucial component during myeloid differentiation) mutations and its relation with chemoresistance in pediatric AML was documented [51,81]. The maternally expressed 3 non-protein-coding gene (*MEG3*), a tumor suppressor, has also been associated with significantly reduced overall survival rate in AML patients. This gene is related to a variety of human tumors and data point out that directly enhance the anticancer effect through p53 [82,83]. Benetatos et al. [53] evaluated the aberrant promoter methylation of *MEG3* in 42 AML patients, and found that *MEG3* hypermethylation was present in 47.6% AML cases and might be associated with significantly reduced overall survival rate in these patients [53]. LncRNAs have also been profiled from AML patients cytogenetically normal (CN) and with specific translocation. For example, AML patients carrying *NPM1*, *CEBPA*, *IDH2*, *ASXL1*, and *RUNX1* mutations and internal tandem duplication mutations in *FLT3* (FLT3/ITD) gene exhibited specific lncRNA expression signature. As well, Diaz-Beya et al. [84], studying AML cases with t(15;17), t(8;21), inv(16), t(6;9), t(3;3), t(9;11), t(8;16), FLT3/ITD, and monosomal karyotype, found a specific lncRNA profile in t(15;17), t(6;9), and t(8;16) positive cases. That study also revealed a correlation between t(8;16) and *linc-HOXA11*, *HOXA11-AS*, *HOTTIP*, and NR_038120 expression, and suggested that GAT2 is an important transcription factor to these lncRNAs. Otherwise, lncRNAs expression correlated with treatment response and survival. One of the lncRNAs that is specifically upregulated in CN-AML cases with *CEBPA* mutation is the lncRNA *UCA1* [85]. Taurine-upregulated gene 1 (*TUG1*) expression was reported to be associated with higher white blood cell counts, monosomal karyotype, FLT3/ITD mutation, and worse prognosis in AML adults. In vitro studies in AML cells indicates that *TUG1* induces cell proliferation but suppressing cell apoptosis via targeting *AURKA* [86].

Schwarzer et al. [44] made a high-density reconstruction of the human coding and non-coding hematopoietic landscape to identify an ncRNA fingerprint associated with lineage specification, HSPC maintenance, and cellular differentiation. They define a core ncRNA stem cell signature in normal HSCs and AML blast, which can serve as a prognostic marker in a different cohort of AML patients and may pave the way for novel therapeutic interventions targeting the non-coding transcriptome [44].

## 8. LncRNAs in Acute Lymphoblastic Leukemia

Data regarding lncRNA playing a role in ALL are still scarce. One of the first clinicopathological correlations with lncRNA expression data in ALL was performed by Fernando et al. [70] who studied 160 children with B-ALL observing that *BALR-2* correlates with overall survival and with response to prednisone. These authors also demonstrated a putative mechanism in regulating cell survival in B-ALL that it is downregulated by glucocorticoid receptor engagement, and that its downregulation results in the activation of the glucocorticoid receptor signaling pathway [70]. Loie et al. [71] also reports that lncRNA expression patterns can classify ALL disease by subtypes as well as protein-coding genes. In addition to lncRNA, *BARL-2*, which is also correlated with resistance to prednisone treatment, these authors found that lncRNAs *BALR-1*, *BRL-6*, and *LINC0098* were overexpressed in pre-B ALL cases and that all of these genes correlated with cytogenetic abnormalities, disease subtypes, and survivals of B-ALL patients [71]. In that study, they also observed that diverse coding genes adjacent to several of those lncRNAs showed unique overexpression profile in *ETV6/RUNX1* positive BCP-ALLS suggesting a possible *cis* regulatory relationship. Furthermore, Ghazavi et al. [47] identified an *ETV6/RUNX1*-specific lncRNA signature in a 64 children cohort and in 13 BCP-ALL cell lines. Five-hundred-and-ninty-six lncRNA transcripts (434 up- and 162 downregulated) showed significant differential expression between *ETV6/RUNX1*-positive BCP-ALL and other genetic BCP-ALL subclasses. However, 16 lncRNAs, of which 14 were upregulated and two were found downregulated, overlapped with the *ETV6/RUNX1*-specific lncRNA signature, including *NKX2-3-1*, *lncRTN4R-1*, *lncGIP-1*, *lnc-LRP8-3*, *lnc-TCF12-2*, *lncC8ort4-1*, *lnc-C8orf4-2*, *lnc-TINAGL1-1*, *lnc-LSM11-4*, and *lnc-SARDH-1* (also known as *DBH-AS1*). *Lnc-SARDH-1* is known to possess an oncogenic role promoting cell proliferation and cell survival through activation of MAPK signaling in the context of hepatocellular carcinoma [87]. Furthermore, the H3K27ac epigenetic mark (associated to enhancers) was found in nine loci of the rest of the lncRNAs and their adjacent coding genes, which, in addition to the finding of a unique expression signature of these coding genes in *ETV6/RUNX1* pre-B ALL, suggests a *cis* interaction between the lncRNAs and their neighboring coding genes [47]. In another study, Ouimet et al. performed a whole transcriptome analysis in a 56 pre-B ALL children cohort finding five lncRNAs specifically overexpressed in pre-B ALL. These genes may have impact in cancer traits such a cell proliferation, migration, apoptosis and treatment response. Specifically, lncRNA *RP11-137H2.4* had a considerable impact on apoptosis, proliferation, and cell migration and its silencing is sufficient to restore a NR3C1-independent cellular response to glucocorticoid (GC) in GC-resistant pre-B ALL cells, leading to GC-induced apoptosis [72]. Further to this study, Gioia et al. functionally characterized three lncRNAs—*RP-11-624C23.1*, *RP11-203E8*, and *RP11-446E9*—specifically repressed in pre-B ALL, restoring their expression in a pre-B ALL cell line. All the lncRNAs promoted tumor suppressor-like phenotypes: apoptosis induction in response to DNA damaging agents and a reduction in cell proliferation and migration [88]. Additionally, Garitano-Trojaola et al., while analyzing ALL samples and peripheral blood samples obtained from healthy donors, found 43 lncRNAs abnormally expressed in ALL. *Linc*-*PINT* was downregulated both in T- and B-ALL cases [89]. Studies in T-ALL cells found a significant difference in expression of *LUNAR1* and *lnc-FAM120AOS-1* between *NOTCH1* wild type and mutant cases [68]. The use of bioinformatics tools identified that *lnc-OAZ3-2:7*—located near the RORC gene—was repressed in this leukemia subtype [90]. These studies suggest that lncRNAs might be utilized as diagnostic and prognostic markers in leukemia, but additional analyses are needed.

## 9. Future Outlooks: Potential Clinical Implications on LncRNAs in Acute Leukemia

It is suggested that more than 97% of the transcribed genome does not encode for proteins. The discovery of the biological role of these non-coding genes took place in 1990, when *XIST* was reported to be involved in X chromosome inactivation (XCI) and gene dosage compensation. Subsequently, *HOTAIR* was identified as a repressor of *HOX* family gene transcription [91]. Most recently, high-throughput expression analyses have been conducted to identify thousands of expressed lncRNA genes either in normal or tumor tissues, showing the potential of lncRNAs as biomarkers for different types of cancer [37,44,52].

Deciphering the molecular mechanisms involved in hematological malignancies addresses new routes to improve diagnosis, prognosis, and treatment of patients with leukemia. In fact, abnormal expression of specific lncRNAs have been reported to be associated with some clinicopathological parameters and molecular subtypes in AL. As example, *BALR-1* and *LINC0098* have been identified as correlating with poor overall survival and diminished response to prednisone treatment in B cell ALL cases [70,71]. Regarding AML, *HOTAIR*, *IRAIN*, and *SNHG5* have been suggested as biomarkers for diagnosis [92]; meanwhile, *UCA1* overexpression was associated with chemoresistance of pediatric cases [81]. *SNHG5* upregulation, which was detected in bone marrow and plasma, was correlated with unfavorable cytogenetics and shorter overall patient survival and was suggested as an independent factor to predict prognosis in AML [93].

Notwithstanding, few of these genes have been replicated across cohorts, probably evidencing biases due to different sample collection and processing techniques, but also as a consequence of AL biological complexity, which is characterized by a wide range of interactions among coding and non-coding genome and spatiotemporal relationships. *HOTAIR*, a proliferation promotor of leukemic blast and leukemia stem cells [94], is one of the most consistently found in AL. A high-expression level defines a subgroup of AL patients with high white blood cell counts at the time of diagnosis and low survival rates [95,96]. Recently, *HOTAIR* high-expression was associated with acquired resistance to antileukemic drugs such as doxorubicin and immatinib [97,98], making this gene as a potential therapeutic target molecule that could contribute to solve a tremendous problem in leukemia chemotherapy, the drug-resistance. On the other hand, experimental data suggest that *HOTAIR* low-expression could be mediated by small interference RNA (siRNA), but still no evidences exist regarding its potential benefit in humans [98]. The development of new molecular strategies as CRISPR/Cas9 to edit the mutated genome or nanotechnology approaches to deliver drugs specifically to leukemia cells prognosticate high applicability of lncRNA as a target to develop new treatments to leukemia [99,100]. Additionally, the high specificity and feasible detection in tissues, serum, plasma, urine, and saliva of the lncRNAs led us to think that lncRNAs could be useful as signals of specific cellular states or read-outs of active cellular pathologies such as leukemia, being promising as predictive biomarkers and potential therapeutic targets in cancer [19].

There is no doubt of the role of lncRNAs in hematopoietic cell transformation, disease evolution, or drug resistance; nevertheless, due to the limited number of studies in hematological entities, these applications are still inconclusive. In fact, before their use as biomarkers in childhood AL, prospective and well-designed cohort studies with adequate sample sizes and further validation of the results in independent cohorts are needed to confirm their clinical usefulness. Therefore, translating this knowledge into the clinical practice still represents a big challenge.

## 10. Conclusions

At this time, we know that lncRNAs are playing a relevant role in cancer development, including leukemia. However, the knowledge regarding molecular mechanisms underlying the pathogenesis of these diseases remains limited. Massive parallel analysis techniques and, likewise, transcriptome expression analysis and RNA sequencing technologies are increasing the possibility to identify those lncRNAs potentially involved in the pathogenesis of AL and other hematopoietic malignancies. To date, large improvements of the surveillance of AL cases have been achieved; nevertheless, cases still die during the AL treatment. Thus, it is necessary to find suitable biomarkers for early diagnosis and accurate risk stratification in AL patients. The association of lncRNAs with several subtypes of leukemia, such as *MEG3*, *IRAIN*, and *UCA1* related to AML and *ANRIL*, *LUNAR1*, in ALL, increase the possibility to use them as biomarkers for the diagnosis, prognosis, and treatment (to provide a target) for the different subtypes of this disease. In addition, further investigation of the function of aberrant expressed lncRNAs may help to understand the pathogenesis of hematological malignancies and provide an important insight in childhood leukemia therapy.

## Figures and Tables

**Table 1:** Classification of lncRNAs according to their function.

Functional Type	Cellular Location	Mechanism of Action	Examples	Reference
Guide	Nucleus	Essential for the proper localization of proteins to their site-specific reaction.	*XIST*, *ANRIL*	[34]
Decoys	Plasma membrane, nucleus and cytosol	Sequestering regulatory factors (transcription factors, catalytic proteins subunits, chromatin modifiers, etc.) to modulate transcription	GAS5, *MALAT1*	[35,36,37]
Scaffold	Nucleus	Providing platforms for assembly of multiple-component complexes such as the polycomb repressive complexes and ribonucleoprotein complex.	*CDKN2B-AS1*, *HOTAIR*	[35,36]
Signaling	Nucleus	Serving as a molecular signal to regulate transcription in response to various stimuli	*TP53COR1*, *PANDAR*	[35,36]
Enhancer	Nucleus	Binding with mediator complex to enhance transcription	*HOTTIP*, *CCAT1-L*, *LUNAR1*	[25,33]

**Table 2:** Examples of lncRNAs described in acute leukemia.

LncRNAs	Classification	Function	Target Genes	Expression Level in Leukemia	Reference
Myeloblastic Leukemia					
*IRAIN*	Intronic	Intrachromosomal interactions	*IGF1R*	Downregulated in leukemia cell lines and in patients with high risk AML	[49]
*UCA1*	Intergenic	Proliferation of AML cells. Oncofetal gene	*CDKN1B*	Upregulated	[50,51,52]
*MEG3*	Intergenic	Tumor suppressor gene	*P53*	Downregulated	[52,53]
*RUNXOR*	Sense	Chromosomal translocations	*RUNX1*	Upregulated	[54]
*NEAT1*	Intergenic	Myeloid differentiation cells	*Unknown in AML*	Downregulated	[50,52,55]
*LLEST*		Tumor suppressor	*BCL-2*	Downregulated or even not expressed.	
*HOTAIRM1*	Antisense	Myeloid differentiation cells, autophagy mechanisms, chromatin remodeling and architecture	*HOXA1*, *HOXA4*, *CD11b* and *CD18*	Upregulated	[52,56,57,58,59,60]
*HOXA-AS2*	Antisense	Apoptotic repressor in NB4 promyelocytic leukemia cells	Unknown	Upregulated	[61]
*PU.1-AS*	Antisense	Involved in the translation of PU.1	*PU.1*	Downregulated	[62]
*WT1-AS*	Antisense	*WT1* expression	*WT1*		[63]
*EGO*	Intronic	*MBP* and *EDN* expression			[41]
*BGL3*	Intergenic	Apoptosis and DNA methylation	*miR-17*, *miR-93*, *miR-20a*, *miR-20b*, *miR-106a* and *miR-106b*	Upregulated	[50,52,64]
*CCAT1*	Intergenic	Monocytic cell differentiation	*miR-155*		[9,52,65]
*CCDC26*	Intergenic	AML cell proliferation	*c-Kit*		[66]
*HOTAIR*	Intergenic	Apoptosis inhibitor	*miR-193a* and *c-Kit*	Upregulated	[67]
*PVT1*	Intergenic	Proliferation of promyelocytes	*MYC*	Upregulated	[52,68]
*ZNF571-AS1*	Antisense	Regulator of JAK/STAT signaling pathway	*KIT* and *STAT5*		[69]
Lymphoblastic Leukemia					
*BALR-2*	Uncharacterized	Unknown	Unknown	Overexpressed in prednisone-resistant B-ALL patients	[70]
*BALR-1*	Unknown	Unknown	Unknown	Upregulated	[70]
*BARL-6*	Unknown	Promotes cell survival and inhibits apoptosis	Unknown	Upregulated	[70]
*LINC00958*	Intergenic	Unknown	Unknown	Upregulated in t(12;21) preB cALL	[70,71]
*DBH-AS1*	Antisense	Cell proliferation and cell survival	Unknown	Upregulated	
*RP11-137H2.4*	Uncharacterized	Apoptosis, proliferation, cell migration	Unknown	Upregulated. Glucocorticoids resistance	[72]
*ANRIL*	Antisense	Cellular proliferation and apoptosis	*CDKN2A/B. CBX7*, *SUZ12*	Upregulated	[52]
*T-ALL-R-LncR1*	Unknown	Promotor of the formation of Par-4/THAP1 protein complex, and the activity of caspase-3	Unknown	Upregulated in children with T-ALL	[73]
*LUNAR1*	Enhancer-like	Promotor of T-ALL proliferation by inducing IGF1R expression.	*IGF1R*	Downregulated	[50,52,74]
*MALAT1*	Intergenic	Alternative splicing and epigenetic modification	Unknown	Upregulated Downregulated in vincristine-resistant ALL	[50,52,75,76,77]
*CASC15*	Intergenic	Cellular survival and proliferation	*SOX4*	Upregulated	[48]

## Abbreviations

1. ABL1	2. ABL protoconcogene 1
3. ABL2	4. ABL protooncogene 2
5. AL	6. Acute leukemia
7. ALL	8. Acute lymphoblastic leukemia
9. AML	10. Acute myeloblastic leukemia
11. ANRIL	12. Antisense non-coding RNA in the INK4-ARF locus B-ALL B cell Acute lymphoblastic leukemia
13. ARID1A	14. AT-rich interaction domain 1A
15. AURKA	16. Aurora kinase A gene
17. BALR	18. B-ALL-associated long non-coding RNAs BL Burkitt Lymphoma
19. CAS9	20. CRISPR associated protein 9
21. CBF	22. Core-binding factor subunit beta
23. CCAT1	24. Colon cancer associated transcript 1 ceRNA Competing endogenous RNA
25. CDKN2A	26. Cyclin dependent kinase inhibitor 2A
27. CDKN2B	28. Cyclin dependent kinase inhibitor 2B
29. CDKN2B-AS1	30. CDKN2B antisense RNA 1
31. CEBPA	32. CCAAT enhancer binding protein alpha
33. CHD6	34. Chromodomain helicase DNA binding protein 6
35. circRNA	36. Circular RNA
37. CLL	38. Chronic lymphocytic leukemia
39. CML	40. Chronic myeloblastic leukemia
41. CN	42. Cytogenetically normal
43. COLDAIR	44. COLD assisted intronic non-coding RNA
45. CREBBP	46. CREB binding protein
47. CRISPR	48. Clustered regularly interspaced short palindromic repeats
49. CRLF2	50. Cytokine receptor like factor 2
51. CSF1R	52. Colony stimulating factor 1 receptor
53. DCs	54. Dendritic Cells
55. DNMT3A	56. DNA methyltransferase 3α
57. EBF1	58. Early B cell factor 1
59. EGO	60. Eosinophil granule ontogeny
61. EP300	62. E1A binding protein P300
63. eRNAs	64. Enhancer RNAs
65. ETS1	66. ETS proto-oncogene 1 transcription factor
67. ETV6	68. ETS Variant6
69. FLC	70. Flowering repressor locus
71. FLT3	72. Fms related tyrosine kinase 3
73. fRNAs	74. Functional RNAs
75. GAS5	76. Growth specific 5
77. GEO	78. Gene expression omnibus
79. H19	80. Imprinted maternally expressed transcript
81. hnRNP	82. Heterogenous nuclear ribonucleoprotein
83. HOTAIR	84. The HOX transcript antisense intergenic RNA
85. HOTTIP	86. HOXA distal transcript antisense RNA
87. IGFR1	88. Insuline-like growth factor type 1
89. IKZF1	90. IKAROS family zinc finger 1
91. IRAIN	92. IGFR1 antisense imprinted non protein RNA
93. ITPR1	94. Inositol1,4,5-triophosphate receptor type 1
95. JAK2	96. Janus kinase 2
97. KIT	98. Tyrosine protein kinase
99. LincRNA	100. Long intergenic non-coding RNA
101. LncRNA	102. Long non-coding RNA
103. lnc-DC	104. Dendritic cell-specifict lncRNA
105. lincRNA-p21	106. Large intergenic non-coding RNA p21
107. lncRNA	108. Long non-coding RNA
109. LUNAR1	110. Leukemia-associated non-coding IGF1R
111. MALAT1	112. Metastasis associated lung adenocarcinoma transcript 1 MCL Mantle cell lymphoma
113. MEG3	114. Maternally expressed 3
115. miRNA	116. MicroRNA
117. mRNA	118. Messenger RNA
119. NCBI	120. National center of biotechnology information
121. ncRNA	122. Non-coding RNA
123. NFAT	124. Nuclear factor activated T cells
125. nmRNA	126. Non messengers RNA
127. npcRNA	128. Non protein-coding RNA
129. NRAS	130. NRAS proto-oncogene
131. NRON	132. Non-protein-coding RNA Repressor of NFAT
133. NSD2	134. Nuclear receptor binding SET domain protein 2
135. PANDAR	136. Promoter of CDKN1A antisense DNA damage activated RNA
137. PAX5	138. Paired box 5
139. PBX1	140. PBX Homeobox 1
141. PCGP	142. Pediatric cancer genome project
143. PDGFRB	144. Platelet derived growth factor receptor beta
145. piRNAs	146. PIWI-interacting RNAs
147. PML	148. Promyelocytic Leukemia gene
149. PROMPTs	150. Promoter-associated long RNAs
151. RB1	152. RB transcriptional corepressor 1
153. RBPs	154. RNA-binding proteins
155. RUNX1	156. Runt related transcription factor 1
157. SHP1	158. Scr homology region 2 domain containing phosphatase-1
159. siRNA	160. Small interference RNA
161. snRNAs	162. Small nuclear RNA
163. snoRNAs	164. Small nucleolar RNA
165. STAT3	166. Signal transducer and activator of transcription 3
167. TCF3	168. Transcription Factor 3Ç
169. TERC	170. Telomerase RNA component
171. TET2	172. Tet methylcytosine dioxygenase 2
173. TLR	174. Tool-like receptor
175. TP53	176. Tumor protein P53
177. TP53COR1	178. Tumor protein P53 pathway corepressor 1
179. TUG1	180. Taurine-up regulated gene 1
181. UCA1	182. Urothelial carcinoma associated 1
183. vlincRNA	184. Very long intergenic RNA
185. XIST	186. X inactive specific transcript
